# Gut microbiota and its derived SCFAs regulate the HPGA to reverse obesity-induced precocious puberty in female rats

**DOI:** 10.3389/fendo.2022.1051797

**Published:** 2022-12-09

**Authors:** Li Wang, Hao Xu, Bin Tan, Qin Yi, Huiwen Liu, Hongrong Deng, Yunxia Chen, Rui Wang, Jie Tian, Jing Zhu

**Affiliations:** ^1^ Department of Pediatric Research Institute, Children’s Hospital of Chongqing Medical University, National Clinical Research Center for Child Health and Disorders, Ministry of Education Key Laboratory of Child Development and Disorders, Chongqing Key Laboratory of Pediatrics, Chongqing, China; ^2^ Department of Clinical Laboratory, Chengdu Women’s and Children’s Central Hospital, School of Medicine, University of Electronic Science and Technology of China, Chengdu, China; ^3^ Department of Clinical Laboratory, Children’s Hospital of Chongqing Medical University, Chongqing, China; ^4^ Department of Cardiovascular Internal Medicine, Children’s Hospital of Chongqing Medical University, Chongqing, China

**Keywords:** obesity, puberty, microbiota, short chain fatty acids (SCFAs), high-fat diet

## Abstract

The intestinal microbiota and its derived short-chain fatty acids (SCFAs) can reverse obesity and obesity-related metabolic diseases, but whether it has an effect on obesity complicated by precocious puberty and its potential mechanism need to be further understood. The purpose of this study was to investigate the effect of the gut microbiota and its derived short-chain fatty acids (SCFAs) on obesity-induced precocious puberty rats and their regulatory mechanisms. We constructed obesity-induced precocious puberty rats using a high-fat diet (HFD) had notable similarity to precocious puberty caused by obesity due to overeating in children. We then added acetate, propionate, butyrate or their mixture to the HFD, and investigated the effect of intestinal microbiota and its derived SCFAs on the hypothalamic-pituitary-gonadal axis (HPGA) in rats with obesity-induced precocious puberty. We found that obesity-induced precocious puberty rats had an early first estrous cycle, increased hypothalamic mRNA expression of Kiss1, GPR54 and GnRH, and early gonadal maturation. Meanwhile, the intestinal microbiota imbalance and the main SCFAs production decreased in the colon. The addition of acetate, propionate, butyrate or their mixture to the HFD could significantly reverse the precocious puberty of rats, reduce GnRH release from the hypothalamus and delay the development of the gonadal axis through the Kiss1–GPR54–PKC–ERK1/2 pathway. Our findings suggest that gut microbiota-derived SCFAs are promising therapeutic means for the prevention of obesity-induced precocious puberty and provide new therapeutic strategies with clinical value.

## Introduction

Overweight and obesity among children has nearly doubled since 1980, making children with obesity a global epidemic and China become the country with the largest number of children with obesity now (1, 2). Several factors contribute to obesity, such as genetic and dietary factors, but the root cause lie in the imbalance between energy intake and consumption that leads to fat accumulation (3, 4). However, children with obesity, the incidence of metabolic diseases such as hyperinsulinemia, dyslipidemia, disturbances of sex hormones, which are significantly related to childhood obesity, are also increasing. The risk of cancer in adulthood is also significantly increased by childhood obesity (5, 6). In recent years, the incidence of precocious puberty in children with obesity has gradually increased. Several studies have shown that high body mass index (BMI) is associated with earlier pubertal maturation in children (7–10). However, it is still unclear whether obesity contributes to precocious puberty. Regarding the relationship between childhood obesity and precocious puberty, researchers believe the key metabolic peptide leptin and genetic factors play important roles (11, 12).

Over the past few years, there has been increasing recognition that the gut microbiota is responsible for regulating obesity and endocrine *via* the gut-brain axis (13–16). A disorder of the gut microbiota and a high proportion of Firmicutes to Bacteroides contributed to childhood obesity (17, 18). By altering their gut microbiota, HFD mothers can promote obesity and precocious puberty in their children (19). Similarly, recent studies have reported that gut microbiota may affect sex hormones and participate in the pathogenesis of precocious puberty in children and HFD mice (18, 20, 21). It is generally known that the initiation of the HPGA and the release of gonadotropin-releasing hormone (GnRH) are necessary for the physiological initiation of precocious puberty. During the activation of GnRH neurons, the gene product kisspeptin encoded by Kiss1 binds to its receptor GPR54 (Kiss1R) stimulate hypothalamic neurons to release GnRH, which promotes the secretion of luteinizing hormone (LH) and follicle stimulating hormone (FSH) in the pituitary (22, 23). However, the specific mechanism by which the gut microbiota regulates the HPGA is still unknown.

Gut microbiota-derived SCFAs also regulate obesity and related metabolic disorders. Biologically, SCFAs are formed from the fermentation of indigestible starch and dietary fiber by intestinal microbes. In colon fecal, SCFAs were primarily composed of acetate, propionate and butyrate at molar ratios of 60:20:20 (24). SCFAs target multiple organs and tissues and regulate related gene expression and tissue metabolism (25, 26). Studies have confirmed that high levels of dietary fiber and SCFAs supplementation in rodents can alleviate HFD-induced obesity (27). However, the specific regulatory role of the gut microbiota and its derived SCFAs on HPGA in obesity-induced rats remains unknown. Herein, we provide evidence that supplementation with SCFAs can reverse and delay the onset of obesity-induced precocious puberty rats by regulating the expression of HPGA related genes.

## Materials and methods

### Animals

Pregnant Sprague−Dawley rats were acquired from Chongqing Medical University (Chongqing, China), which were housed under standard conditions: light and dark cycles for 12 hours and temperature 22 °C. Upon birth, the female rats were weaned on postnatal day (PND) 21. Subsequently, all the female animals were randomly assigned to different experimental groups after PND21 (n= 6-8). The control group was allowed free access to pelleted food (CTR; Diet D12450B, 10% fat content, Keao Xieli, China) and tap water. The HF group was fed a high-fat diet (HF; Diet D12451, 45% fat content, Keao Xieli, Beijing, China). The rats in the SCFAs groups received supplementation with 5% sodium acetate (HF-A, S2289, Sigma-Aldrich, USA), sodium propionate (HF-P, P1880, Sigma-Aldrich, USA) or sodium butyrate (HF-B, 303410, Sigma-Aldrich, USA), and the mixed group (HF-SCFA) received sodium acetate, sodium propionate or sodium butyrate at a ratio of 3:1:1 in the high-fat diet (Diet D12451, 45% fat content, Keao Xieli, Beijing, China). Body weight was monitored daily. All experiments were approved by the Animal Ethics Committee of Children’s Hospital of Chongqing Medical University.

### Phenotypic evaluation of pubertal maturation

Indicators for evaluating the maturity of female pubertal rats. Monitoring puberty by observing the vaginal opening (VO) after PND21. Exfoliated cells from the rats with vaginal opening were examined by smear to confirm first estrus. The rats were sacrificed at diestrus stage of the estrous cycle, while the remaining rats were sacrificed at the same time. We used histological analysis and scoring method of ovary (Pub-Score) to analyze the development of follicles and corpus luteum (CL) according to the reference (28, 29). For non-ovulating animals, determine the most advanced healthy antral follicle class from small follicles to antral follicles (F1-F5), thus determining the time of prepubertal maturity from -5 to-1 (representing the time of the first ovulation). For rats that ovulated, the CL was used as the morphological sign of ovulation, allowing puberty to be divided from +1 (equivalent to CL1) to +5 (equivalent to CL5) at intervals of 1 day.

### Sample preparation

All rats were anesthetized by intraperitoneal injection of 2% pentobarbital sodium. Blood samples were collected from the abdominal aorta, and the serum was separated after centrifugation (3500 rpm, 20 min, 4°C) and stored at -80°C. The serum hormone and blood lipid levels were measured. After sacrifice, the hypothalamus, pituitary, uterus, ovary, colon and fecal were removed, frozen in liquid nitrogen and preserved at -80°C. The wet weight of the uterus and ovary was determined, relative uterus weight (UW) and ovary weight (OW) (mg/100 g) were calculated. The ovary and uterine tissues were stained with formalin for hematoxylin and eosin (H&E) staining.

### Hormone assays

The concentrations of serum FSH, LH and GnRH were measured by ELISA kits (Jianglaibio, China). The minimum detection concentrations of FSH and LH were less than 0.38 mIU/ml and 0.074 mIU/ml, respectively. Hypothalamic tissue was weighed, and precooled PBS was added according to the weight–volume ratio (1:9). After the homogenate was fully ground on ice, the supernatant of the homogenate was centrifuged (5000 g, 10 min). The minimum detection concentration of GnRH was less than 7.9 pg/ml. All samples were measured in duplicate. For all hormones, the intra-and inter-batch coefficients were less than 10%.

### Detection of blood lipids

All samples were measured for triglycerides, total cholesterol, low-density lipoprotein cholesterol (LDL-C), and high-density lipoprotein cholesterol (HDL-C) by standard laboratory techniques using a Hitachi automatic analyzer (Hitachi 7600, Japan).

### Real-time PCR analysis

Using TRIzol reagent (Takara, Japan), total RNA was obtained from the rat hypothalamus, pituitary, colon and ovary. One microgram of total RNA was reverse transcribed into cDNA using Superscript II reverse transcription kit (Takara, Japan). Then, cDNA obtained by reverse transcription and gene-specific primers and a SYBR Green dye kit (Takara, Japan) were used for amplification. **Table 1** shows the forward and reverse primers used for the polymerase chain reaction. β-actin was used as the endogenous control. The amount of mRNA relative to the endogenous control was calculated by the 2^-△△CT^ method.

**Table 1:** Primers used for qPCR analyses.

Target gene	Forward Sequence (5’→3’)	Reverse Sequence (5’→3)’
*GnRH*	CGCTGTTGTTCTGTTGACTGTGTG	TCCTCCTCCTTGCCCATCTCTTG
*Kiss1*	GCTGCTGCTTCTCCTCTGTGTG	GACTGTTGGCCTGTGGGTTCAG
*GPR54*	CTTTCCTTCTGTGCTGCGTACCC	CGAGACCTGCTGGATGTAGTTGAC
*ERα*	TCCTCCTCATCCTTTCCCATATCCG	GCATCTCCAGCAGCAGGTCATAG
*GnRHR*	GCTGCCTGTTCATCATCCCTCTTC	GCTGTAGTTTGCGTGGGTCCTG
*LHR*	TAACGAGACGCTTTATTCCGCCATC	AGCATCTGGTTCTGGAGCACATTG
*FSHR*	GGTCTCCTTGCTGGCATTCTTGG	CGGAATCTCTGTCACCTTGCTGTC
*GPR43/FFAR2*	GGCTGTGGTGACGCTTCTTAATTTC	GGCTGGCATTGAGGGAACTGAAC
*GPR41/FFAR3*	TGCCTCTACACCATCTTCCTCTTCC	CCGCTGCCAGGTTGATGAAGTAC
*GPR109a*	CCCGCACCACTTCCTGAACAAG	CCGTGTAGAGGAGGTGGACTGTC
ACTB	AGATCAAGATCATTGCTCCTCCT	ACGCAGCTCAGTAACAGTCC

### Western blot analysis

Rat hypothalamus proteins were extracted using protein extraction kit (Beyotime, China). Protein samples were separated to 10% SDS−PAGE gels. After electrophoresis, the proteins were transferred to polyvinylidene fluoride (PVDF) membranes (Millipore, USA). The membranes were blocking for 1 hour, then incubated overnight at 4°C with primary antibodies against GPR54 (A2967, ABclonal, Shanghai, China), PKC (2056T, Cell Signaling, USA), phospho-ERK1/2 (9101s, Cell Signaling, USA), ERK1/2 (9102S, Cell Signaling, USA), and β-actin (TA-09, China). Next, the membranes were incubated with the corresponding secondary antibody (ZSGB-BIO, China) at 37°C for 1 hour. The membranes were visualized by chemiluminescent substrate (Millipore, USA) under the ChemiDOC Touch Imaging System (Bio-Rad, USA). The results were analyzed by ImageJ software.

### 16S rDNA

Fecal DNA was extracted from different samples using OMEGA stool DNA Kit according to the manufacturer’s instructions. The V3-V4 regions per sample were amplified using primers by a PCR system (GeneAmp 9700, ABI, USA). Amplicons were paired-end sequenced with the Illumina NovaSeq 6000 (Illumina, USA) by LC-Bio Technology Co., Ltd. (Hangzhou, China). Raw fastq files were merged using FLASH and high-quality filtered using fqtrim. Chimeric sequences were filtered by Vsearch. After dereplication using DADA2, we obtained a feature table and feature sequence. Alpha-diversity and β-diversity were calculated by QIIME. The ASVs were annotated by aligned feature sequences with the SILVA database. Other diagrams were implemented using the R package.

### SCFA quantification

Detection and analysis of SCFAs in rat fecal samples. Briefly, acetic acid, propanoic acid, butyric acid, isobutyric acid, isovaleric acid, hexanoic acid and valeric acid were mixed with ether at different volumes to prepare the standard concentration gradient. Then, 50 mg of each sample was obtained, the internal standard acetic-d4 was added, and methanol was added to 0.5 ml. The samples were ultrasonicated for 10 min in an ice bath, vortexed and centrifuged (12,000 rpm, 4°C, 5 min). A total of 1μl was injected and detected by gas chromatography-mass spectrometry (Thermo Trace1610, USA).

### Statistical analysis

All the results are presented as the mean ± standard deviation. Two-paired Student’s *t*-*test* was used for two group, one-way analysis of variance (ANOVA) followed by Duncan or Turkey comparison was used for multiple group comparisons. The Wilcoxon rank-sum test was used for the analysis of microbial difference. And the data were plotted by GraphPad Prism 8.0 Software (GraphPad Software Inc., USA). *P* < 0.05 indicated a statistically significant difference.

## Results

### Effects of the HFD on the pubertal development of immature female rats

**Figure 1:**
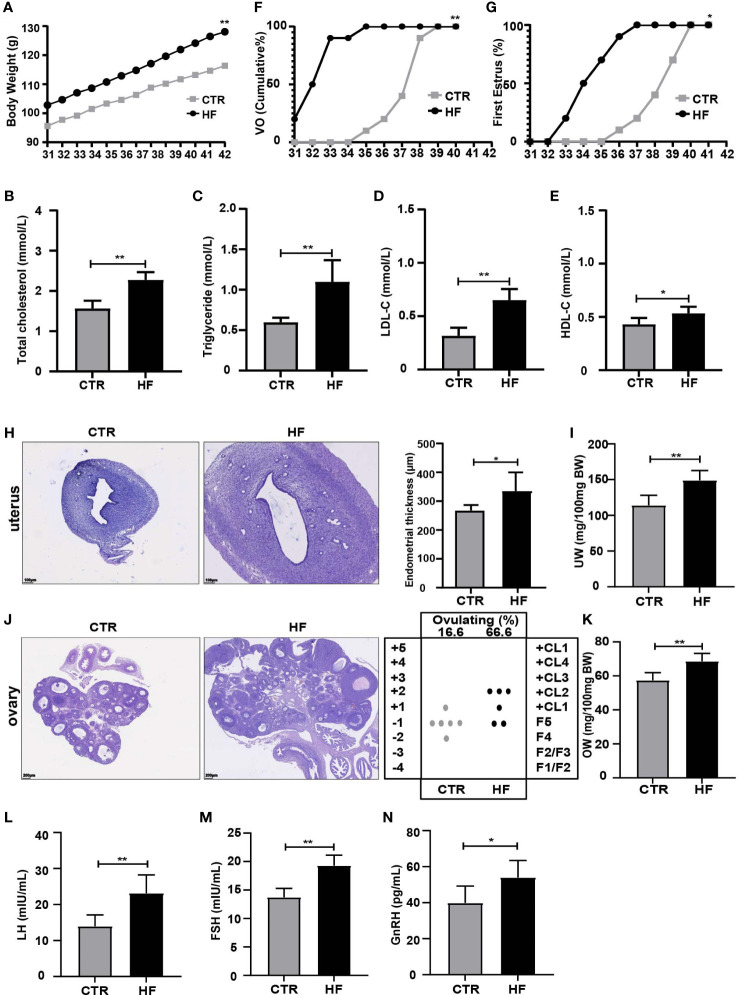
HFD induces obesity and precocious puberty in female rats. **(A)** Evolution of body weight. **(B)** Serum total cholesterol. **(C)** Serum triglyceride. **(D)** Serum low density lipoprotein cholesterol (LDL-C). **(E)** Serum high density lipoprotein cholesterol (HDL-C). **(F)** Cumulative percentage of vaginal opening. **(G)** Cumulative percentage of first estrus. **(H)** Uterine wall thickness. **(I)** Relative uterus weight. **(J)** Histological score of follicular development and ovulation. **(K)** Relative ovary weight. **(L)** Serum LH levels. **(M)** Serum FSH levels. **(N)** GnRH secretion in the hypothalamus. n = 6 per group for rat samples. *P<0.05; **P<0.01.

Female rats fed a HFD to construct an obesity model had an increased body weight (BW) during the peripubertal period (**Figure 1A**), and female adolescent rats had increases in total cholesterol, triglycerides, LDL-C and HDL-C (**Figures 1B–E**). Furthermore, precocious puberty was induced in obese rats, as characterized by the earlier age VO (**Figure 1F**) and first estrous (**Figure 1G**). At the same time, endometrial thickness (**Figure 1H**) and UW (**Figure 1I**) were increased. We found that the HF group had a higher percentage undergone ovulation according to the histological score of the ovary (66.66% HF versus 16.6% CTR) (**Figure 1J**) and OW (**Figure 1K**). Our results also showed that the relative release of serum LH (**Figure 1L**) and FSH (**Figure 1M**) and the secretion of GnRH from the hypothalamus (**Figure 1N**) were significantly increased in the HF group. Altogether, these results support that HFD leads to obesity and consequently induces precocious puberty in rats.

### Obesity-induced precocious puberty promotes the gene expression of the HPGA

**Figure 2:**
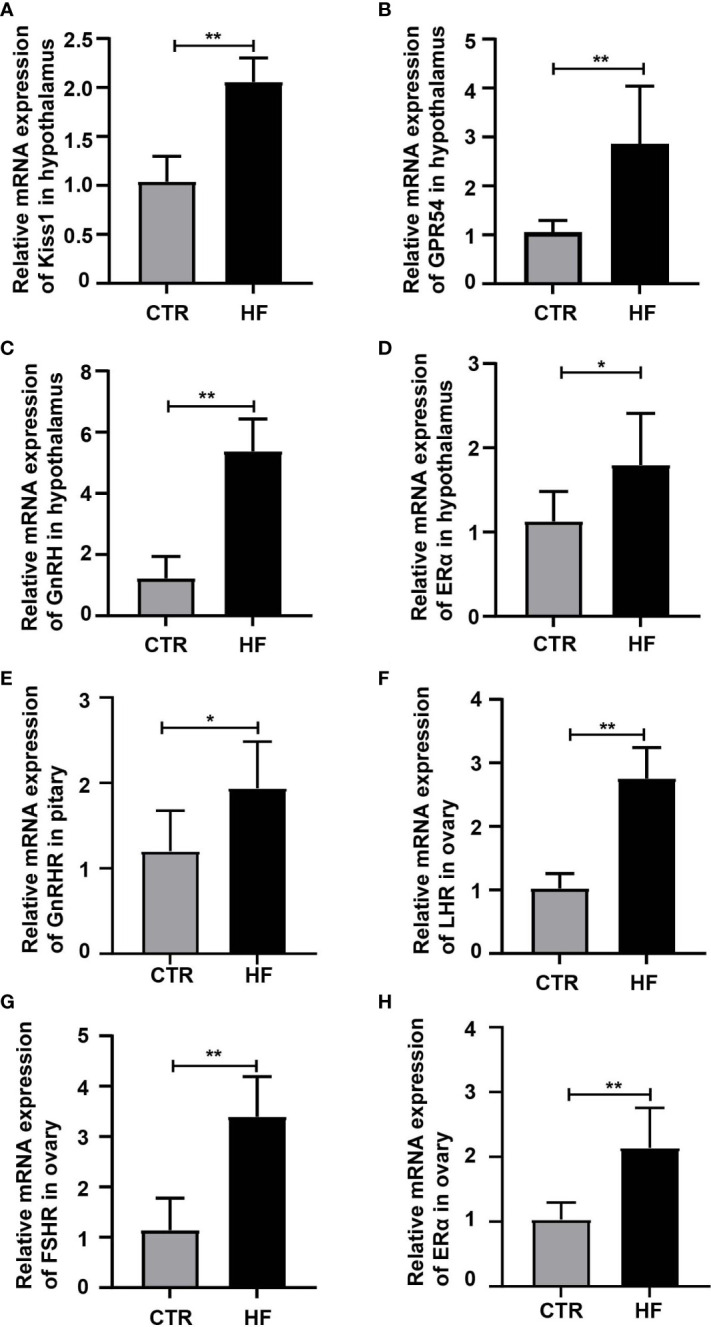
HFD promotes the gene expression of the HPGA in rats with obesity-induced precocious puberty. **(A)** The gene expression of Kiss1 in the hypothalamus. **(B)** The gene expression of GPR54 in the hypothalamus. **(C)** The gene expression of GnRH in the hypothalamus. **(D)** The gene expression of ERα in the hypothalamus. **(E)** The gene expression of GnRHR in the pituitary. **(F)** The gene expression of LHR in the ovary. **(G)** The gene expression of FSR in the ovary. **(H)** The gene expression of ERα in the ovary. n = 6 per group for rat samples. *P<0.05; **P<0.01.

To further explore the effects of HFD-induced obesity on the HPGA, we first detected the key genes regulating the gonadal development pathway. The results showed that the expression of *Kiss1*, *GPR54*, *GnRH* and *ERα* in the hypothalamus were higher than in the CTR group (**Figures 2A–D**), which in turn activated the gonadotropin-releasing hormone receptor (GnRHR, **Figure 2E**) in the pituitary, and the luteinizing receptor (LHR, **Figure 2F**), follicle-stimulating hormone receptor (FSHR, **Figure 2G**) and estrogen receptor α (ERα, **Figure 2H**) in the ovary. The above results showed that obesity-induced precocious puberty promotes the gene expression of the HPGA.

### Obesity-induced precocious puberty alters gut microbiota in female rats

**Figure 3:**
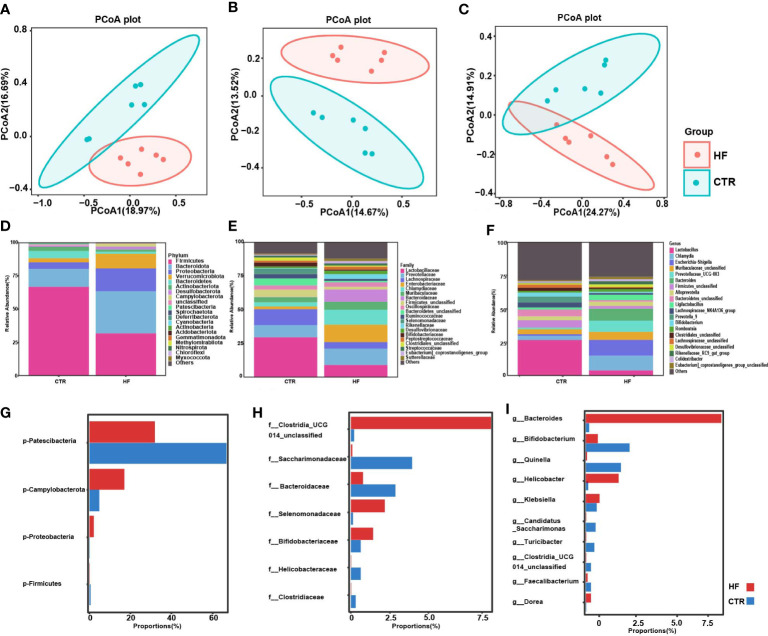
HFD alters gut microbiota in obesity-induced precocious puberty female rats. During postnatal day (PND) 21, female rats were fed a HFD and a normal diet until the rats were sacrificed during diestrus after completing the estrous cycle. Microbial 16S rDNA genes were subsequently sequenced. **(A)** PCoA plot of Bray-Curtis distance of the gut microbiota. **(B)** PCoA plot Jaccard distance of gut microbiota. **(C)** PCoA plot of unweighted Uni-Frac distance of gut microbiota. **(D–F)** Community composition distribution at the phylum, family and genus level. **(G–I)** The most differentially abundant proportions between the HF and CTR group at the phylum, family and genus levels. n=6 per group for gut microbiota analysis. Significance was tested with the Wilcoxon rank-sum test.

To explore the changes in the gut microbiome in rats with obesity-induced precocious puberty, we analyzed the feces between the HF group and CTR group. The composition of the microbiota in the feces of the HF group was significantly different from that of the CTR group (**Figure 3**). Based on Bray−Curtis, Jaccard and unweighted Uni-Frac PCoA, there was a significant difference in the β-diversity of the feces between the HF group and CTR group (**Figures 3A–C**). At the phylum level, compared with those in the CTR group, the HF group showed increased levels of Bacteroidota, decreased levels of Firmicutes, and increased Proteobacteria and Verrucomicrobiota(**Figure 3D**). However, the proportion of Patescibacteria was significantly lower and the levels of Campylobacterota and Proteobacteria were higher than that in the CTR group (*P < 0.05*, **Figure 3G**). At the family level, the levels of *Lactobacillaceae* and *Lachnospiraceae* were decreased in the HF group, while the level of *Enterobacteriaceae* was increased (**Figure 3E**). The proportions of *Clostridia*, *Selenomonadaceae* and *Bifidobacteriaceae* in the HF group were significant higher. And the level of *Saccharimonadaceae*, *Helicobacteraceae* and *Clostridaceae* were significantly lower than CTR group *(P < 0.05*, **Figure 3H**). At the genus level, the HFD induced a decrease in *Lactobacillus* and an increase in *Chlamydia* in HF group (**Figure 3F**). The proportions of *Bacteroides*, *Helicobacter* and *Klebsiella* showed a significant increase, and the proportions of *Bifidobacterium*, *Quinella* showed a significant decrease (*P < 0.05*, **Figure 3I**). These data suggest that the abundance of gut microbes in obesity-induced precocious puberty rats was significantly different from that in CTR group.

### Obesity-induced precocious puberty rats change the level of fecal SCFAs

**Figure 4:**
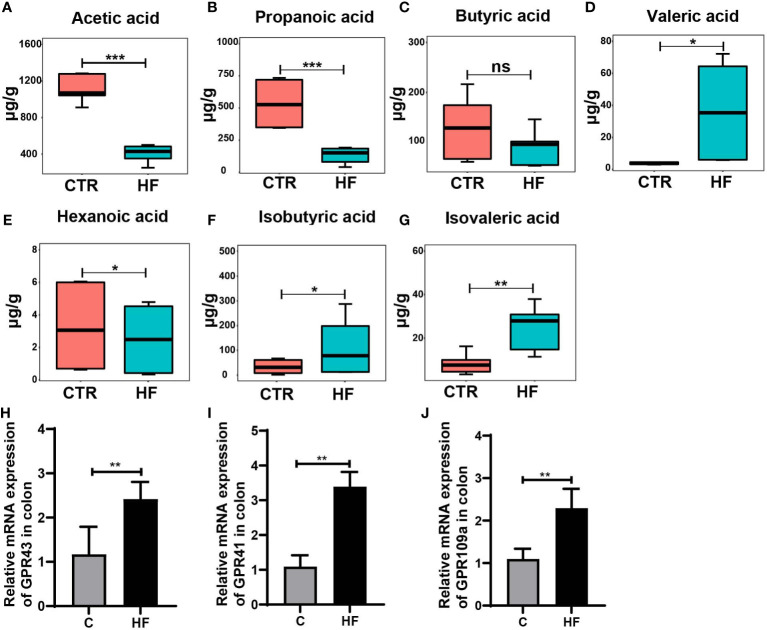
HFD alters the levels of fecal SCFAs and their related receptors in the colon in the rats with obesity-induced precocious puberty. **(A)** The content of acetic acid in fecal. **(B)** The content of propanoic acid in fecal. **(C)** The content of butyric acid in fecal. **(D)** The content of valeric acid in fecal. **(E)** The content of hexanoic acid in fecal. **(F)** The content of isobutyric acid in fecal. **(G)** The content of isovaleric acid in fecal. **(H)** The gene expression of GPR43 in the colon. **(I)** The gene expression of GPR41 in the colon. **(J)** The gene expression of GPR109a in the colon. Fecal SCFAs levels were detected by GC-MS. n = 6 per group for rat samples. *P<0.05; **P<0.01; ***P<0.001. ns, not significant.

It is widely recognized that the intestinal microbiota affects the endocrine system by synthesizing neurotransmitters and active metabolites, such as SCFAs (30). In our study, the SCFAs levels in the feces of obesity-induced precocious puberty rats were significantly changed. Our results showed that the levels of SCFAs in the HF group were significantly lower in acetic, propionic and hexanoic acid. (**Figures 4A, B**, **Figure 4E**). Although the level of butyric acids was not statistically different, there was a trend to decrease (**Figure 4C**). Correspondingly, the levels of valeric acid, isobutyric acid and isovaleric acids increased (**Figures 4D**–**G**). Furthermore, the mRNA expression of *GPR43*, *GPR41* and *GPR109a* in the colon (**Figures 4H–J**) was significantly increased in the HF group.

### SCFAs reverse obesity and precocious puberty in obesity-induced precocious puberty rats

**Figure 5:**
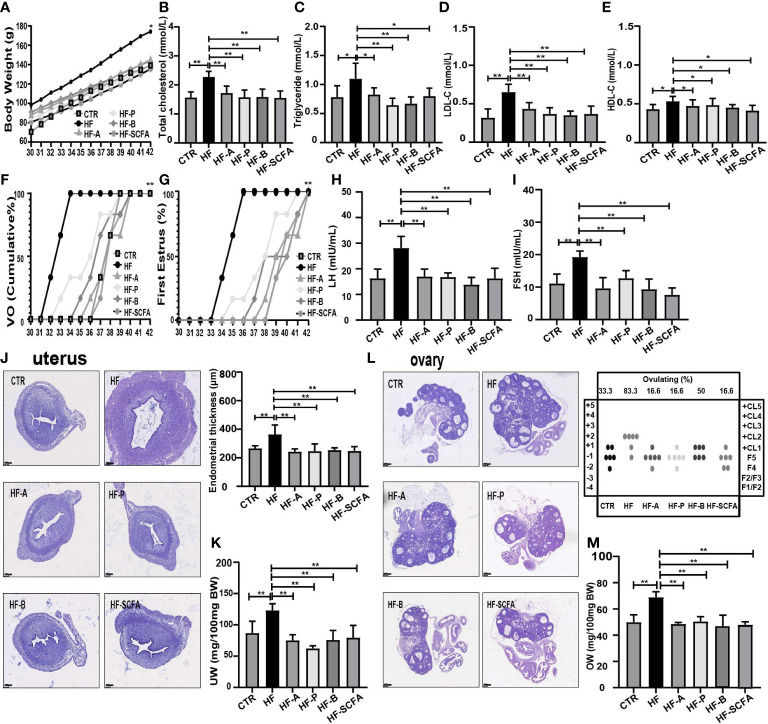
SCFAs reverse obesity and precocious puberty in obesity-induced precocious puberty rats. **(A)** Evolution of body weight. **(B)** Serum total cholesterol. **(C)** Serum triglyceride. **(D)** Serum low density lipoprotein cholesterol (LDL-C). **(E)** Serum high density lipoprotein cholesterol (HDL-C). **(F)** Cumulative percentage of vaginal opening. **(G)** Cumulative percentage of first estrus. **(H)** Serum LH levels. **(I)** Serum FSH levels. **(J)** Morphological and histopathological changes in uterine and uterine wall thickness after addition of SCFAs. **(K)** Relative uterus weight. **(L)** Histological score of follicular development and ovulation after addition of SCFAs diet. **(M)** Relative ovary weight. n = 6 per group for rat samples. *P<0.05; **P<0.01.

To further explore whether obesity-induced precocious puberty is caused by intestinal microbiota and SCFAs, we added 5% sodium acetate, sodium propionate or sodium butyrate or the three together as a mixture to the HFD. After adding the SCFAs sodium acetate, sodium propionate or sodium butyrate or their mixture, the body weight of immature female adolescent rats was significantly lower than that of the HF group (**Figure 5A**). Compared to the HF group, supplementation with SCFAs attenuated HFD-induced total cholesterol, triglyceride, LDL-C and HDL-C levels (**Figures 5B–E**). In addition, the age of VO and the first estrous cycle were significantly delayed (**Figures 5F, G**). In HFD rats that received diet supplementation with SCFAs, there was a significant decrease in serum LH and FSH (**Figures 5H, I**).

### SCFAs inhibit the development of sexual organs in rats with obesity-induced precocious puberty

After supplementation with 5% SCFAs in HFD, histopathological and morphological changes in the uterus showed that the thickness of the uterine endometrium (**Figure 5J**) in the SCFAs treated groups was obviously lower than that in the HF group, as was the relative uterus weight (UW) (**Figure 5K**). To further assess the ovarian physiology of different groups, we evaluated the histological score of follicular development and ovulation. As shown in **Figures 5L, M**, SCFAs reduced the percentage of rats that ovulated by the HFD that reached ovulation (HF 83.3% versus HF-A 16.6% versus HF-P 16.6% versus HF-B 50% versus HF-SCFA 16.6%), as well as the relative ovary weight (OW). The above studies show that SCFAs can significantly affect the development of sexual organs in rats.

### SCFAs reverse the expression of genes related to the HPGA in rat with obesity-induced precocious puberty

**Figure 6:**
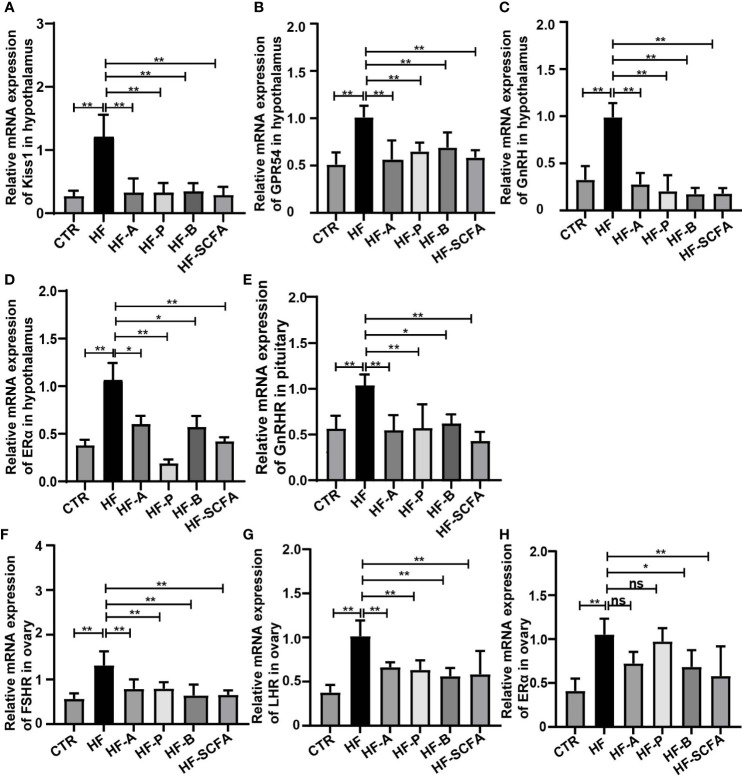
SCFAs reverse the expression of genes related to the HPGA in rats with obesity-induced precocious puberty. **(A)**The gene expression of Kiss1 in the hypothalamus. **(B)** The gene expression of GPR54 in the hypothalamus. **(C)** The gene expression of GnRH in the hypothalamus. **(D)** The gene expression of ERα in the hypothalamus. **(E)** Gene expression of GnRHR in the pituitary. **(F)** The gene expression of FSR in the ovary. **(G)** The gene expression of LHR in the ovary. **(H)** The gene expression of ERα in the ovary. n = 6 per group for rat samples. *P<0.05; **P<0.01.

We observed the effect of SCFAs on the gene expression of the HPGA. Compared with the HF group, 5% sodium acetate, sodium propionic, sodium butyrate and their mixtures significantly reduced the mRNA expression of the *Kiss1, GPR54, GnRH* and *ERα g*enes in the hypothalamus (**Figures 6A–D**). Considering the pituitary responsiveness to hypothalamic neuropeptides, the levels of *GnRHR* were found to significantly decline with the addition of SCFAs (**Figure 6E**). LH, FSH and estrogen control the overall activity of the female reproductive tract by binding to their receptors on ovarian cells (31). To this end, we detected *LHR*, *FSHR* and *ERα* in the ovary. Consistent with the development of the ovary, SCFAs reduced the expression of the gonadotropin receptors *LHR*, *FSHR* and *ERα* in the ovary (**Figures 6F–H**). The above results indicate that SCFAs significantly reduce HFD-mediated changes in the expression of genes related to HPGA, thereby restoring its level to that of a normal diet.

### SCFAs change the gut microbiota in rats with obesity-induced precocious puberty

**Figure 7:**
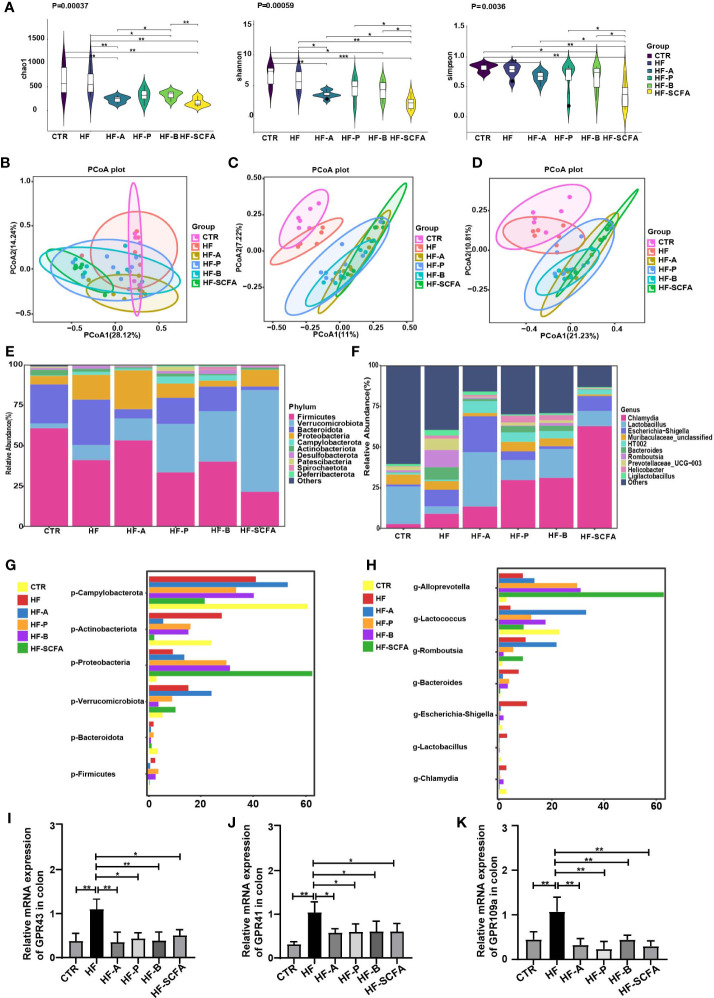
SCFAs regulate microbiota and its receptors change in obesity-induced precocious puberty rats. During PND21, female rats were fed HFD, and 5% acetate, propionate, butyrate diet and their mixtures were added until the rats were sacrificed during diestrus after completing in estrous cycle. Microbial 16S rDNA genes were subsequently sequenced. **(A)** α-diversity of the chao1, shannon and simpon index in different groups. **(B)** PCoA plot of Bray-Curtis distance of the gut microbiota. **(C)** PCoA plot Jaccard distance of gut microbiota. **(D)** PCoA plot of unweighted Uni-Frac distance of gut microbiota. **(E, F)** Community composition distribution at the phylum and genus levels. **(G, H)** The most differentially abundant proportions between the HF, CTR, HF-A, HF-P, HF-B and HF-SCFA groups at the phylum and genus levels. n=6-8 per group for gut microbiota analysis. Significances was tested with the Wilcoxon rank-sum test. **(I)** The gene expression of GPR43 in the colon. **(J)** The gene expression of GPR41 in the colon. **(K)** The gene expression of GPR109a in the colon. n = 6 per group for rat samples. *P<0.05; **P<0.01.

We further examined the intestinal microbiota of rats in the HF the group and SCFAs intervention groups. We found that the fecal α-diversity analysis based on Chao1, Shannon and Simpson indexes in the SCFAs intervention groups revealed a significant decrease compared with the HF group (**Figure 7A**). Analysis of β-diversity based on the Bray−Curtis, Jaccard and unweighted UniFrac PCoA analyses showed that the microbiota of the SCFAs added groups were significantly distinct (**Figures 7B–D**). This was consistent with previous studies showing that HFD increases gut microbiota biodiversity (20). SCFAs reverse the biodiversity of HFD. Notably, at the phylum level, there were significant differences in Firmicutes, Bacteroidota, Verrucomicrobiota, Proteobacteria, Actinobacteriota and Campylobacterota (**Figures 7E**, **G**). Interestingly, after adding the SCFAs mixture, compared with that in the HF group, the bacterial change in relative abundance was the most significant. At the genus level, there were significant differences among *Chamydia* and *Lactobacillus*, *Escherichia-Shigella*, *Bacteroidetes*, *Romboutsia*, *Lactococcus* and *Alloprevotella* (**Figures 7F**, **H**). The above data showed that compared with the HF group, the abundance of gut microbiota changed significantly after SCFAs intervention. Considering the protective effect of SCFAs on the metabolic parameters studied, as well as on the change in gut microbiota, we evaluated the SCFAs binding and activation the orphan G-protein-coupled receptors *GPR43*, *GPR41* and *GPR109a* in the colon. The elevated mRNA levels of *GPR43*, *GPR41* and *GPR109a* (**Figures 7I–K**) showed a significant decrease in SCFAs-supplemented groups compared to the HF group.

### SCFAs regulate hypothalamic GnRH release through the GPR54–PKC–ERK1/2 pathway

**Figure 8:**
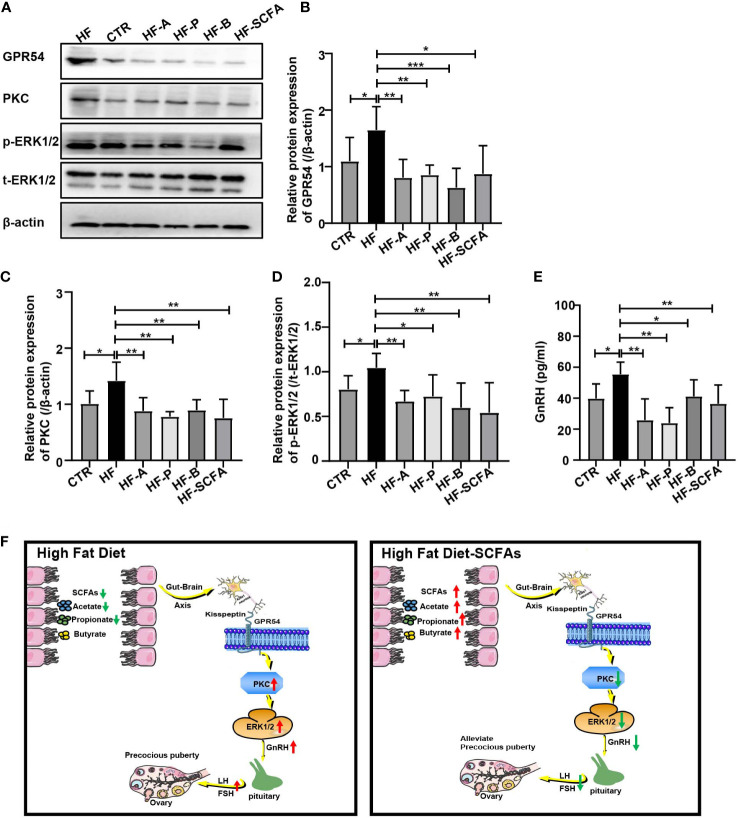
SCFAs regulate hypothalamic GnRH release through GPR54-PKC-ERK1/2 pathway. **(A)** Representative Western blot images showing GPR54, PKC, p-ERK1/2, t-ERK1/2 and β-actin expression in rats added with SCFAs. **(B–D)** Statistical analysis of GPR54, PKC and p-ERk1/2 expression in each group. **(E)** GnRH secretion in the hypothalamus. **(F)** HFD led to a decrease in SCFAs in the gut and promoted the expression of the GPR54-PKC-ERK1/2 pathway. Supplementation with SCFAs with a HFD reversed the high expression of the GPR54-PKC-ERK1/2 pathway and further decreased the secretion of GnRH, weakening the activation of the pituitary gland and inhibiting gonadal development. n = 6 per group for rat samples. *P<0.05; **P<0.01; ***P<0.001.

Studies have reported that GnRH release may be related to PKC and phosphorylated ERK1/2, as phosphorylation of these proteins is considered the main cellar signaling event in the activation of the GPR54-GnRH pathway (32, 33). To further explore the potential molecular regulatory mechanism of gut microbiota-derived SCFAs of the GPR54-GnRH signaling pathway in the hypothalamus, we measured the protein levels of *GPR54*, *PKC*, and *ERK1/2* in the hypothalamus (**Figure 8A**) and examined the secretion of GnRH in the hypothalamus between the HF group and the SCFAs supplement groups. Compared with the HFD, SCFAs significantly reduced the protein expression of *GPR54* and *PKC* and the phosphorylation of *ERK* (**Figures 8B–D**) and the secretion of GnRH (**Figure 8E**) in the hypothalamus. These data suggest that *GPR54*, *PKC* and *ERK1/2* may be the key mediators of SCFAs in obesity-induced precocious puberty (**Figure 8F**).

## Discussion

Central precocious puberty (CPP) refers to the appearance of secondary sexual characteristics before the age of 8 in girls and 9 in boys. The underlying reason is the premature activation of the HPGA (34). However, the incidence of CPP tends to higher in children with obesity, and accumulative evidence has shown that the intestinal microbiota can change the expression of genes and their related metabolites through SCFAs, which play an important role in weight regulation in children with obesity (35, 36). The concentrations of propionic acid and butyric acid in the feces of children with obesity are lower than those of normal-weight children. This change is related to an imbalance in the abundance of phylum Firmicutes (37). Consistent with previous reports, we found that the obesity-induced precocious puberty rats showed gut microbiota imbalance. Correspondingly, the proportion of gut microbiota-derived SCFAs is also dysregulated (20, 38). In our study, acetic acid, propionic acid and butyric acid in the intestinal tract decreased in obesity-induced precocious puberty rats, and the changes in SCFAs may be related to the changes of *Bacteroides*, *Bifidobacterium*, *Quinella*, *Helicobacter*, *Klebsiella*.

Acetic acid, propionic acid and butyric acid are important components of SCFAs, accounting for more than 95% of SCFAs. Acetate, propionate and butyrate act as synthetic metabolic substrates and signal molecules in cell function (39–42). To further explore the effect of SCFAs on obesity-induced precocious puberty, we used a HFD containing 5% SCFAs to interfere with an obesity-induced precocious puberty rat model and found that SCFAs could significantly reverse the phenotype of obesity, which was consistent with previous results (25). At the same time, we found that SCFAs could also reverse the age of VO and the first estrous cycle as well as endometrial thickness and premature ovarian maturation in obesity-induced precocious puberty rats. The above results imply that SCFAs can alleviate HFD induced obesity and precocious puberty, and that SCFAs supplementation may play a role in the prevention of obesity and precocious puberty.

During the process of puberty induction, hypothalamic Kiss1 and its receptor GPR54 function as important regulators of GnRH nerve cell activation and GnRH secretion and play an important role in puberty maturation (22). Our results confirmed that SCFAs can significantly decrease the gene expression of *Kiss1, GPR54, GnRH* and *ERα* in the hypothalamus of obesity-induced precocious puberty rats. In our study, under the low expression of the *Kiss1* gene and *GPR54*, which ultimately downregulated *GnRHR* activation and secretion of LH and FSH in the pituitary gland. Furthermore, the expression of *LHR* and *FSHR* in the ovaries was reduced, which further delayed the maturation of the ovaries and uterus, reversed the development of the gonadal axis and alleviated the symptoms of precocious puberty in obesity-induced precocious puberty rats. The above results suggest that the interdependence of gut microbiota with SCFAs may be the reason for precocious puberty in HFD.

After adding 5% SCFAs to the HFD, we found that the gut microbiota also changed, accompanied by reversing the symptoms of precocious puberty. SCFAs increased the relative abundance of *Chlamydia* and *Lactobacillus* and decreased the relative abundance of *Bacteroides*, *Romboutsia*, *Lactococcus* and *Alloprevotella*. Consistent with previous studies, the increase in *Chlamydia* is associated with obesity-related metabolic syndrome, and the increase in *Lactobacillus* can reverse obesity induced by HFD and regulate the microbiological SCFAs hormone pathway to regulate metabolism (43, 44). The increase in *Bacteroides* abundance is related to the level of estradiol (18). *Bacteroides*, *Romboutsia*, *Lactococcus* and *Alloprevotella* are related to obesity and related metabolic syndrome and showed a positive correlation with *Lactococcus* and GnRH, and the above bacteria may play a role in promoting sexual development (20, 45, 46). In our results, the increase in *Lactobacillus* and the decrease in *Bcateroides*, *Romboutsia*, *Lactococcus* and *Alloprevotella* may be the reasons why SCFAs reverse HFD-induced precocious puberty in rats. However, changes in intestinal microbiota and SCFAs led to changes of *GPR43*, *GPR41* and *GPR109a* in the colon. It was found that *GPR43*, *GPR41* and *GPR109a* in the intestinal tract of obesity-induced precocious puberty rats were significantly increased, and SCFAs supplementation decreased the expression of *GPR43*, *GPR41* and *GPR109a* in the intestine. The above conclusions are consistent with the conclusions reported in the literature on obesity in mice (25). SCFAs alter the bacterial community structure in the gut and may be responsible for the changes in the expression of *GPR43*, *GPR41* and *GPR109a* (25).

Human GPR54 plays a role in the regulation of endocrine function. Kisspeptin, the product of Kiss1 neurons, binds to GPR54 and stimulates PIP2 hydrolysis, Ca^2+^ mobilization, arachidonic acid release, ERK1/2 and p38 MAP kinase phosphorylation and inhibits cell proliferation (22, 47). To confirm the exact effect of SCFAs on the GPR54-GnRH pathway in obesity-induced precocious puberty rats induced by HFD, we studied its effect on related proteins. We found that the SCFAs in HFD could significantly downregulate the levels of GPR54, reduce the expression of PKC, inhibit phosphorylated ERK1/2 signal pathway, and reduce the secretion of hypothalamic GnRH, thus reducing the secretion of pituitary LH and FSH, thus inhibiting the development of the gonadal axis and relieving the symptoms of precocious puberty.

## Conclusion

These findings indicate the complexity of the mechanism behind obesity-induced precocious puberty. We demonstrated that the gut microbiota and its derived SCFAs play a certain role in the pathogenesis and prevention of obesity-induced precocious puberty. In this complex context, our findings focus on the fact that we act on Kiss1 neurons and their receptor GPR54 through SCFAs derived from intestinal microbiota, and then reduce the release of hypothalamic GnRH and pituitary LH and FSH through the PKC-ERK1/2 pathway and delay the development of the ovary and uterus. As discussed here, the opportunity and improvement of SCFAs for obesity-induced precocious puberty promise to provide new strategies and open avenues for treatment.

## Data availability statement

The datasets generated for this study are included in the article. The data presented in the study are deposited in the NCBI repository, accession number PRJNA893237.

## Ethics statement

The animal study was reviewed and approved by Ethics Committee of Children’s Hospital of Chongqing Medical University (No. CHCMU-IACU20211028002).

## Author contributions

LW carried out all experiments and data analysis and prepared the manuscripts. JZ, JT, BT, and HX designed the experiments, review, and revised the manuscripts. QY and RW contributed to reagent management. HL, YC and HD edit the chart. All authors approved the final manuscript.
